# Supplementary material for: The interactome of intact mitochondria by cross-linking mass spectrometry provides evidence for coexisting respiratory supercomplexes
Source: Mol Cell Proteomics. 2017 Dec 8;17(2):216–32. doi: 10.1074/mcp.RA117.000470 (PMC5795388; doi:10.1074/mcp.RA117.000470)
Supplement: Supplemental Data [file supp_RA117.000470_133922_0_supp_23978_fzffwf.zip › spectra_annotation/mito_spectra_annotation/70-1-2-1-4-1.pdf]

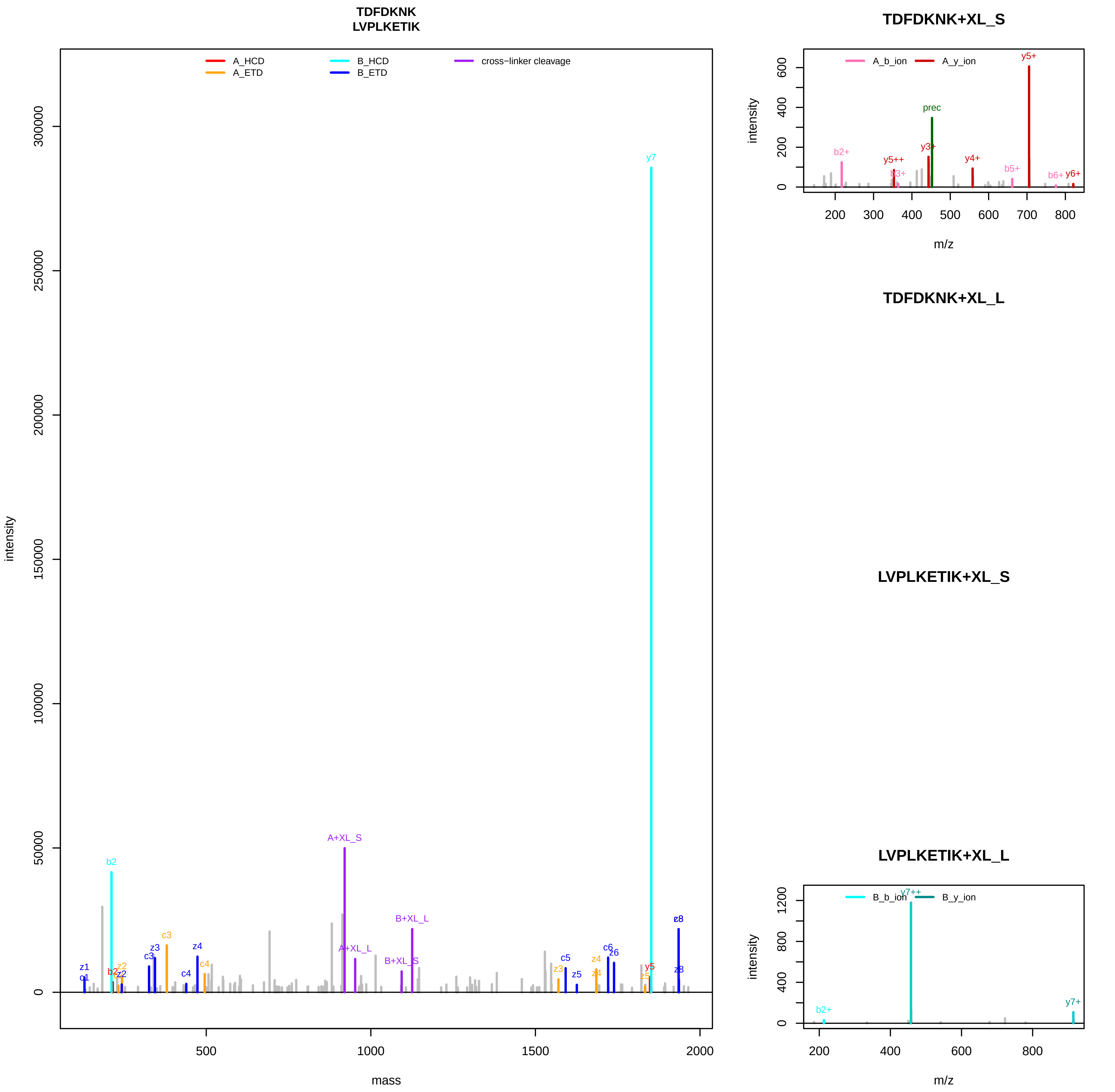

TDFDKNK
LVPLKETIK
A_HCD
A_ETD
B_HCD
B_ETD
cross−linker cleavage
intensity
mass
0
50000
100000
150000
200000
250000
300000
500
1000
1500
2000
y7
b2
A+XL_S
B+XL_L
A+XL_L
B+XL_S
z1
c1
b2
c2
z2
c3
z3
c3
c4
z4
c4
z3
c5
z5
z4
z4
c6
z6
y5
z5
c8
z8
z8
TDFDKNK+XL_S
A_b_ion
A_y_ion
intensity
m/z
0
200
400
600
200
300
400
500
600
700
800
y5+
prec
b2+
y5++
b3+
y3+
y4+
b5+
b6+
y6+
TDFDKNK+XL_L
LVPLKETIK+XL_S
LVPLKETIK+XL_L
B_b_ion
B_y_ion
intensity
m/z
0
400
800
1200
200
400
600
800
y7++
b2+
y7+
